# Experimental Study on Surface Polishing of SLM-316L Stainless Steel via Laser Treatment and Mechanical Grinding

**DOI:** 10.3390/mi16060634

**Published:** 2025-05-27

**Authors:** Wei Fang, Qiuling Wen, Jiaxin Hu, Feng Jiang, Zhongwei Hu, Xian Wu, Jinlin Yang, Xiaoguang Wang

**Affiliations:** 1 Institute of Manufacturing Engineering, Huaqiao University, Xiamen 361021, China; 22014080018@stu.hqu.edu.cn (W.F.); 23014080039@stu.hqu.edu.cn (J.H.); jiangfeng@hqu.edu.cn (F.J.); huzhongwei@hqu.edu.cn (Z.H.); 22014080099@stu.hqu.edu.cn (J.Y.); 22013080036@stu.hqu.edu.cn (X.W.); 2 State Key Laboratory of High Performance Tools, Xiamen 361021, China; 3 College of Mechanical Engineering and Automation, Huaqiao University, Xiamen 361021, China; xianwu@hqu.edu.cn

**Keywords:** SLM-316L stainless steel, nanosecond laser polishing, mechanical grinding, surface roughness

## Abstract

The 316L stainless steel material boasts exceptional corrosion resistance and plasticity, among other benefits, and finds extensive application in automotive components, molds, aerospace parts, biomedical equipment, and more. This work focuses on the surface polishing of selective laser melting (SLM) 316L stainless steel using 1064 nm nanosecond laser processing and mechanical grinding. The influence of laser processing parameters on the surface roughness of SLM-316L stainless steel was investigated using an orthogonal experiment. After laser processing, the surface roughness of SLM-316L stainless steel was reduced from 7.912 μm to 1.936 μm, but many randomly distributed irregular micro-cracks appeared on the surface. EDS and XRD detections illustrated that iron oxides were generated on the surface of SLM-316L stainless steel after laser processing. Mechanical grinding was further performed to achieve a nanometer surface finish and remove the metal oxides and micro-cracks generated on the surface of SLM-316L stainless steel after laser processing. The AFM measurement results indicate that the surface roughness of SLM-316L stainless steel was reduced to approximately 3 nm after mechanical grinding. Moreover, the micro-cracks and iron oxides on the surface of laser-processed SLM-316L stainless steel were completely removed. This work provides guidance for the precision polishing of SLM-316L stainless steel.

## 1. Introduction

In recent years, selective laser melting (SLM) has emerged as an up-and-coming technique in metal additive manufacturing. This technology melts metal powder by scanning a laser beam layer by layer, which enables the fabrication of complex structural parts with high dimensional accuracy, high densities, and high performance [1]. 316L stainless steel, an austenitic stainless steel, is composed of iron, chromium, nickel, and molybdenum. It is known for its excellent corrosion resistance, plasticity, toughness, and reducibility. Therefore, 316L stainless steel is widely used in automotive parts, molds, aerospace components, and biomedical equipment [2,3,4]. The yield strength and surface hardness of SLM-316L stainless steel have been demonstrated to be significantly better than 316L stainless steel produced by conventional rolling methods [4,5,6]. However, the parts produced by SLM technology generally have large surface roughness [7], which does not meet the requirements for directly applying precision parts. Therefore, surface polishing is a necessary process for SLM-316L stainless steel.

Laser polishing is a non-contact surface polishing technology with the advantages of non-pollution, a wide range of processing objects, stable polishing quality, easy-to-realize automation, and so on [8,9,10,11]. The principle of laser polishing is to use the high heat generated by the laser to make the surface layer of the material melt or evaporate. The flow of molten metal is propelled in the molten state by capillary or thermo-capillary forces. The molten state of the metal flows from the peaks to the valleys, creating a relatively flat surface and ultimately a polished material surface [12,13,14]. Therefore, lasers are well suited for the surface polishing of metals, and the metals currently reported in the literature for laser polishing include tool steel [15,16,17], mold steel [18,19,20], titanium alloys [21,22,23], and other metal materials. Zeng et al. [21] reported that the surface roughness of TC4 alloys was reduced from 13.764 μm to 1.756 μm by nanosecond laser polishing with appropriate parameters. Liang et al. [23] showed that the surface roughness of Ti6Al4V can be reduced from 10.2 μm to 2.1 μm by using a nanosecond pulsed laser polishing with suitable parameters. Li et al. [24] employed a nanosecond laser to polish 304 stainless steel with an original roughness of 7.805 μm, resulting in a surface roughness down to 1.879 μm. Lan et al. [25] investigated nanosecond laser polishing of 316L stainless steel. By optimizing the laser polishing parameters, the surface roughness of 316L stainless steel was reduced from 4.84 μm to 0.65 μm. It can be seen from the above literature that although lasers can be used for polishing various metals, such as 316L stainless steel, the surface roughness of stainless steel polished by lasers cannot reach the nanometer-level finish.

In this paper, nanosecond laser polishing and mechanical grinding were combined to achieve precision polishing of SLM-316L stainless steel. The influence of laser defocusing amount, laser power, laser scanning speed, laser scanning times, and laser scanning pitch on the surface quality of SLM-316L stainless steel was investigated. The optimal parameters for laser processing were explored. In addition, the three-dimensional (3D) morphology, micro-morphology, and material composition analysis of SLM-316L stainless steel after laser processing and mechanical grinding were characterized. Through process optimization, the surface roughness of stainless steel can be reduced from 7.9 μm to about 3 nm.

## 2. Materials and Methods

**Figure 1:**
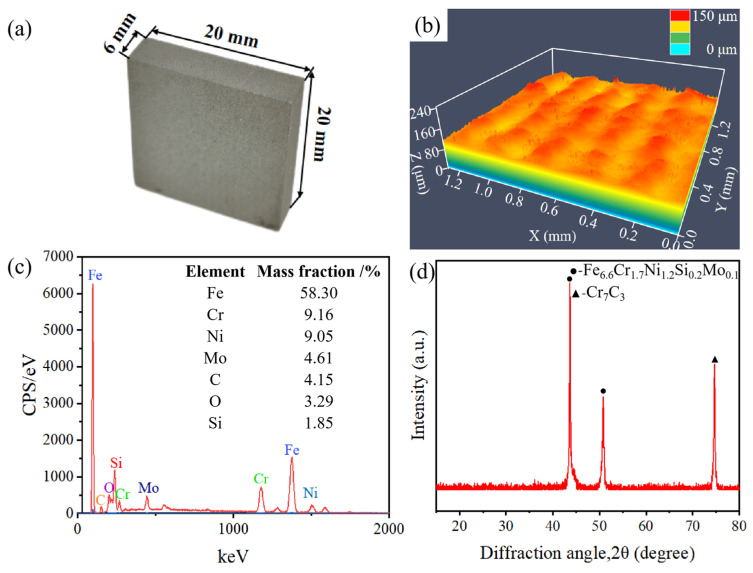
(**a**) Optical photograph, (**b**) surface morphology, (**c**) EDS spectrum, and (**d**) XRD pattern of the original SLM-316L stainless steel.

The SLM-316L stainless steel samples with dimensions of 20 × 20 × 6 mm were provided by Xiamen Wuxinglong Technology Co., Ltd. (Xiamen, China). as shown in Figure 1a. The initial average surface roughness (Sa) of the sample was 7.912 μm, as presented in Figure 1b. The elements of SLM-316L stainless steel are characterized by energy-dispersive spectroscopy (EDS), and the results are displayed in Figure 1c. EDS analysis shows that the elemental composition in weight percentage (wt%) of SLM-316L stainless steel was Fe 58.30%, Cr 9.16%, Ni 9.05%, Mo 4.61%, C 4.15%, O 3.29%, and Si 1.85%. Clearly, the most dominant element in SLM-316L stainless steel is Fe. It is worth noting that the presence of the O element is from the atmosphere. The X-ray diffractogram (XRD) spectrum of the initial SLM-316L stainless steel is shown in Figure 1d. The diffraction peaks at 2θ of 43.66° and 50.82° correspond to Fe_6.6_Cr_1.7_Ni_1.2_Si_0.2_Mo_0.1_ according to PDF No. 50-1293. The diffraction peak located at 2θ = 74.72° corresponds to Cr_7_C_3_ (PDF No. 11-0550). This indicates that the original SLM-316L stainless steel is composed of Fe_6.6_Cr_1.7_Ni_1.2_Si_0.2_Mo_0.1_ and Cr_7_C_3_.

**Figure 2:**
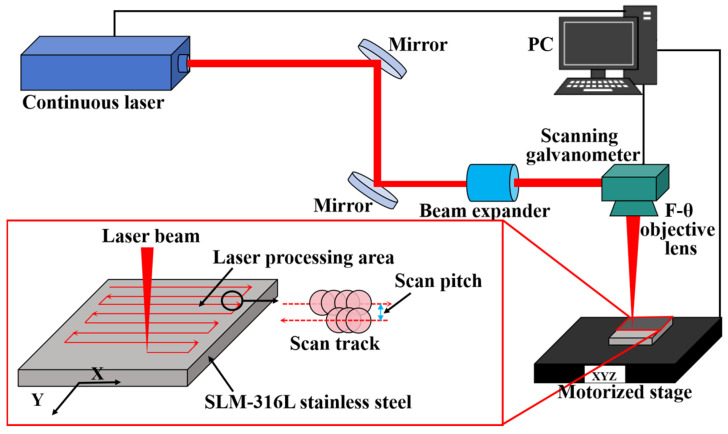
Schematic diagram of the laser processing experimental setup and illustration of the laser scanning strategy.

Before laser processing experiments, SLM-316L stainless steel was ultrasonically cleaned in anhydrous ethanol for ten minutes and then dried with nitrogen. The SLM-316L stainless steel sample was then mounted on a computer-controlled X-Y-Z stage. The laser processing equipment is provided by Century Raymond (Beijing) Technology Co., Ltd. (Beijing, China). The laser source is a nanosecond laser with a working wavelength of 1064 nm, a pulse duration of 2 ns, a repetition rate of 850 kHz, and a maximum power of 20 W. As illustrated in Figure 2, the nanosecond laser beam is first guided by two mirrors into a beam expander and then goes into the galvanometer scanner, and finally focuses normally onto the SLM-316L stainless steel surface through an F-Theta objective lens with a focal length of 160 mm. The focused laser spot diameter is 60 μm. The laser scanning track is shown in the bottom left of Figure 2. The laser processing is performed in an atmospheric environment. In order to verify the repeatability of the test results, a total of three repeatability verification tests were conducted.

The surface roughness of the initial sample and laser-processed sample was measured by laser confocal microscopy (SLM700, Zeiss, Oberkochen, Germany) under identical conditions. The dimensions of the corresponding measurement area are 1.2 mm × 1.2 mm. The surface micro-cracks and metal oxide layer of the SLM-316L stainless steel caused by laser processing were removed using a grinding machine (Tegramin-25, Struers, Copenhagen, Denmark). The surface micro-morphology and elemental composition of SLM-316L stainless steel before and after laser processing and mechanical grinding were characterized using an emission scanning electron microscope (SEM, Apreo S, Thermo Fisher Scientific, Waltham, MA, USA) equipped with EDS. The Sa value of the sample after mechanical grinding was measured using atomic force microscopy (AFM, Alpha300RA, WITec GmbH, Ulm, Germany) under identical conditions. The measurement area of AFM is 5 μm × 5 μm. The material composition of SLM-316L stainless steel before and after laser treatment and mechanical grinding was examined by XRD (D8 Advance, Bruker AXS GmbH, Karlsruhe, Germany).

## 3. Results and Discussion

### 3.1. Influence of Laser Processing Parameters on Surface Roughness of SLM-316L Stainless Steel

An orthogonal experiment was used to optimize the laser processing parameters of SLM-316L stainless steel. Here, the orthogonal design only considers and analyzes the main effects according to Refs. [26,27,28]. We designed a five-factor, four-level orthogonal experiment with laser defocusing amount (*H*), laser power (*P*), laser scanning times (*S*), laser scanning speed (*V*), and laser scanning pitch (*D*) as independent variables. The laser processing parameter settings are shown in Table 1. And the selection of the laser processing parameter range was based on the results of the preceding single-factor experiments. Sixteen groups of processing strategies were tested on the SLM-316L stainless steel samples, and the results of the surface roughness of laser-processed SLM-316L stainless steel are displayed in Table 2. It is important to note that the surface roughness values for SLM-316L stainless steel are averages taken from multiple sets of measurements. The “Expt. No.” column in Table 2 represents the experimental group with different laser processing parameters.

**Figure 3:**
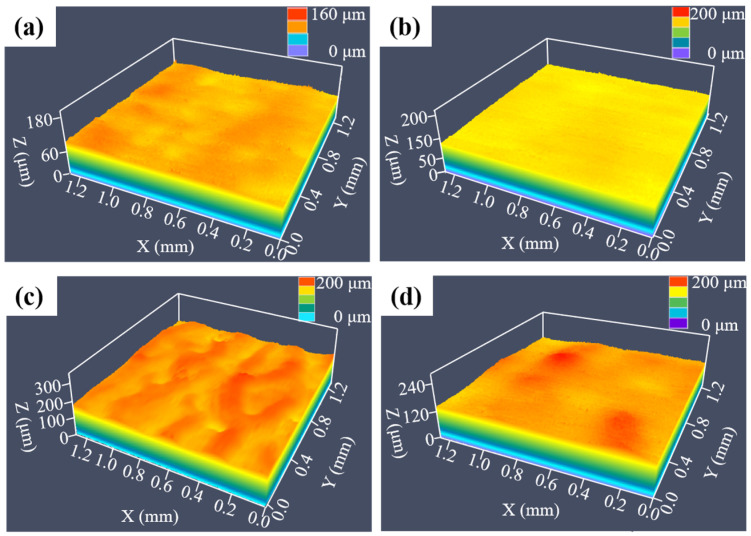
Surface morphologies of SLM-316L stainless steel after laser processing with different laser parameters: (**a**) No. 1, (**b**) No. 8, (**c**) No. 10, and (**d**) No. 13.

Figure 3 displays four representative surface morphologies of laser-processed SLM-316L stainless steel using different laser processing parameters. Comparing the surface morphology of the original sample in Figure 1b, the surface roughness values of Nos. 1, 8, 10, and 13 were reduced to some extent after laser processing. It is noteworthy that No. 8 has the lowest surface roughness value of 1.936 μm, a reduction of 75.5% compared to the untreated SLM-316L stainless steel. This demonstrates that the optimal laser processing parameters are a 0.5 mm laser defocusing amount, 14 W laser power, 100 mm/s laser scanning speed, 8 μm laser scanning pitch, and 25 laser scanning times.

The range analysis method is widely used to assess the weights of the influencing factors because of its low computational complexity. In order to investigate the influence of the weights of laser defocusing amount, laser power, laser scanning times, laser scanning speed, and laser scanning pitch on the surface roughness of SLM-316L stainless steel, we performed a range analysis on the results in Table 2. The results of the range analysis are shown in Table 3. Here, *K_i_* (*i* = 1, 2, 3, 4) in Table 3 represents the mean value of surface roughness for the same level of the factor, and *R* in Table 3 is the difference between the maximum and minimum values of *K_i_*. In the range analysis, the *R* value is calculated as the difference between the maximum and minimum values at each level. The larger the *R* value, the greater the difference in the results, indicating a more substantial impact of that factor on the outcome. Conversely, the smaller the *R* value, the less significant the factor’s influence on the outcome [28]. Therefore, from Table 3, it can be concluded that the significant influences on the surface roughness of laser-processed SLM-316L stainless steel rank in the order of the laser defocusing amount, laser power, laser scanning pitch, laser scanning speed, and laser scanning times. The experimental data in Table 2 were also analyzed using Analysis of Variance (ANOVA) with surface roughness as the response variable. The results are shown in Table 4. As indicated in Table 4, the F-values for laser defocusing amount, laser power, and laser scanning spacing are all greater than 0.15. Therefore, the effects of these three parameters on the Sa value of SLM-316L stainless steel are highly significant. The results of the variance analysis are consistent with the results of the range analysis.

### 3.2. Surface Morphology and Material Composition Analysis of SLM-316L Stainless Steel After Laser Processing

**Figure 4:**
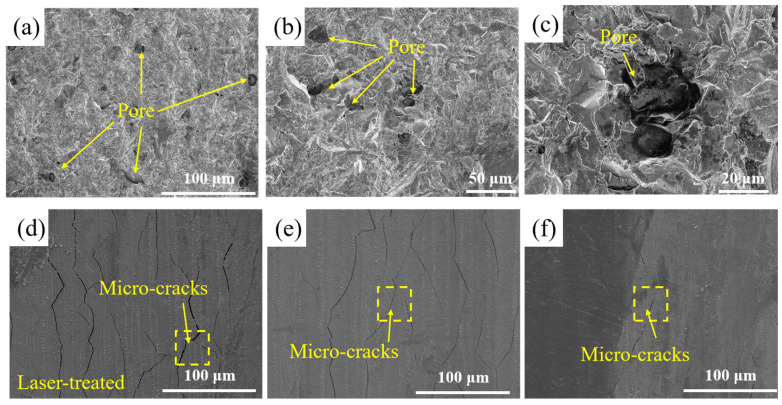
Surface micro-morphologies of (**a**–**c**) original and (**d**–**f**) laser-treated SLM-316L stainless steel.

**Figure 5:**
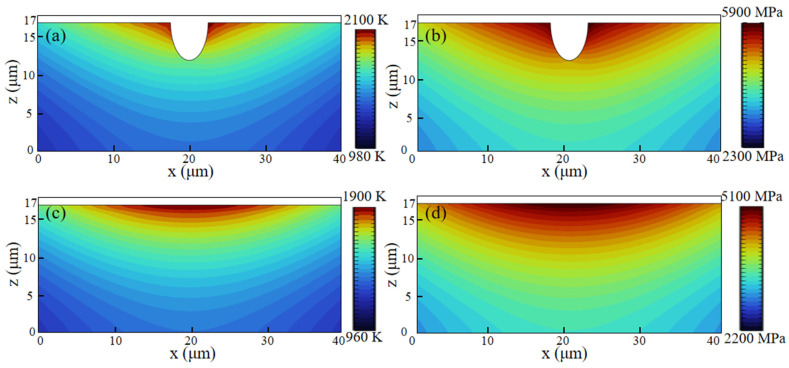
Temperature field and thermal stress distribution of laser irradiation of SLM-316L stainless steel (**a**,**b**) with a defect and (**c**,**d**) without a defect at 140 μs.

The original surface of the SLM-316L stainless steel was extremely uneven, with numerous pores and other defects, as illustrated in Figure 4a–c. As shown in Figure 4d–f, the SLM-316L stainless steel surface after laser treatment is covered with many randomly distributed cracks. To further investigate the impact of surface defects in the original SLM-316L stainless steel on laser polishing, the temperature field and thermal stress during the laser polishing of SLM-316L stainless steel were simulated using the ANSYS 2023 R1 finite element simulation software. The physical properties of SLM-316L stainless steel used in the simulation model are shown in Table 5. In order to simplify the calculation process, the simulation model is simplified to a two-dimensional model. The effect of temperature variation on material properties is disregarded. The size of the simulation model is set to 100 μm × 20 μm. The defect is represented by a semi-ellipse, with the major axis and minor axis of the ellipse measuring 5 μm and 10 μm, respectively. In meshing, all mesh shapes are set as free triangle meshes, where the maximum cell mesh size is 0.67 μm and the minimum cell mesh size is 3 nm, with a total of 11,530 meshes, and mesh refinement is carried out at defects to improve computational accuracy. The boundary conditions are the same as those in Ref. [29]. The input laser fluence is 0.58 J/cm^2^. Figure 5 illustrates the temperature field and thermal stress distribution of SLM-316L stainless steel with and without a defect for a 140 μs duration. It can be seen that the maximum temperature of the SLM-316L stainless steel at the defect location reaches 2100 K, and the maximum thermal stress reaches 5900 MPa, which is obviously larger than those without a defect (1900 K; 5100 MPa). This indicates that the defects in the SLM-316L stainless steel can significantly increase the absorption of laser energy, resulting in a higher temperature and greater thermal stress at the defect location. As a result, micro-cracks are formed in the vicinity of the defect. This finding is consistent with the results reported in Refs. [30,31].

**Figure 6:**
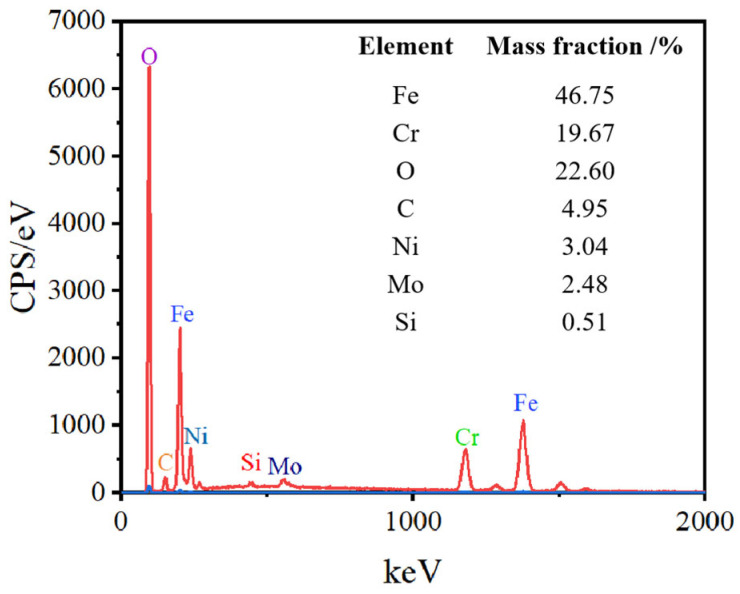
EDS elemental analysis of SLM-316L stainless steel after laser processing.

The EDS elemental analysis of SLM-316L stainless steel after laser processing is shown in Figure 6. The elements of laser-processed SLM-316L stainless steel are the same as those of the original SLM-316L stainless steel, whereas the O content increased from 3.29% to 22.6% after laser treatment. The increase in O content may be due to the oxidation reaction of metal elements in SLM-316L stainless steel with oxygen in the air during the laser irradiation.

**Figure 7:**
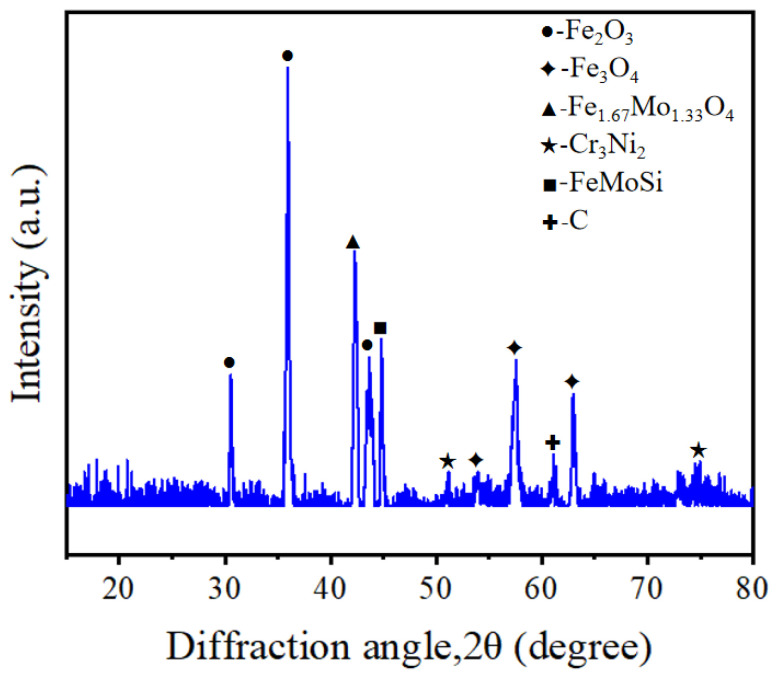
XRD pattern of SLM-316L stainless steel after laser processing.

In order to determine whether the surface of SLM-316L stainless steel after laser processing contains metal oxides, we performed XRD inspection, and the results are shown in Figure 7. The diffraction peaks located at 2θ = 30.54°, 35.94°, and 43.64° correspond to Fe_2_O_3_ according to PDF No. 25-1402. The diffraction peaks located at 2θ values of 53.96°, 57.52°, and 63.00° correspond to Fe_3_O_4_ (PDF No. 26-1136). The diffraction peaks at 2θ values of 42.26° correspond to Fe_1.67_Mo_1.33_O_4_ (PDF No. 42-0324). This confirms that large amounts of iron oxides were produced on the surface of SLM-316L stainless steel after laser processing. In addition, the diffraction peaks located at 51.18° and 74.96° correspond to Cr_3_Ni_2_ (PDF No. 26-0430). The two diffraction peaks at 2θ values of 44.80° and 61.10° correspond to FeMoSi and C, respectively (PDF Nos. 15-0485 and 46-0944). This indicates that the material composition of laser-processed stainless steel consists of Cr_3_Ni_2_, FeMoSi, and C in addition to metal oxides.

### 3.3. Mechanical Grinding of Laser-Treated 316L Stainless Steel

**Figure 8:**
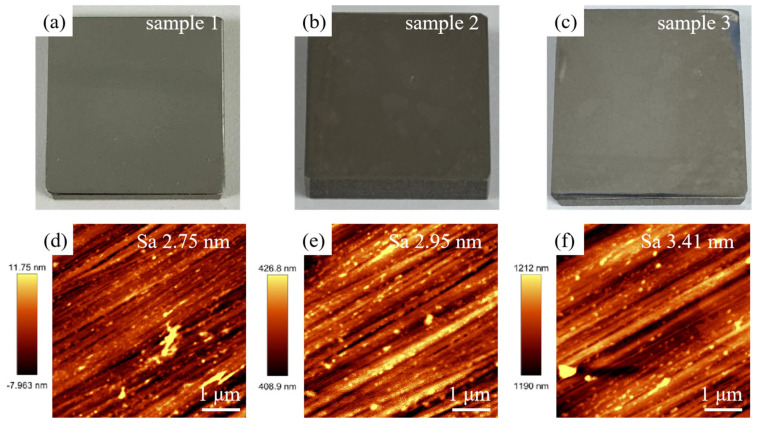
(**a**–**c**) Optical photographs and (**d**–**f**) AFM images of three SLM-316L stainless steel samples after mechanical grinding.

**Figure 9:**
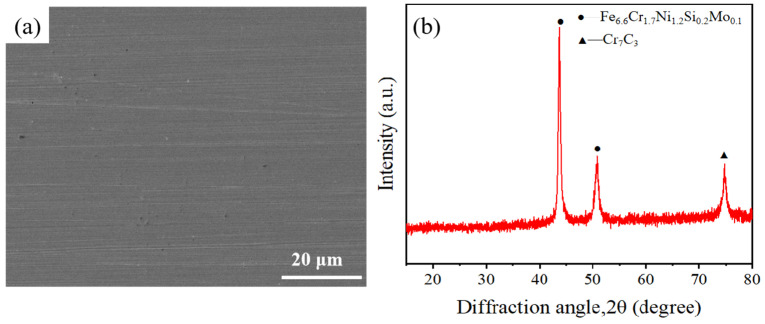
(**a**) SEM image and (**b**) XRD spectrum of SLM-316L stainless steel after mechanical grinding.

After laser treatment, the Sa value of SLM-316L stainless steel is about 1.936 μm, which still fails to meet the application requirements. Moreover, the surface of SLM-316L stainless steel was oxidized, and there were many randomly distributed micro-cracks on the surface. Therefore, mechanical grinding was carried out to achieve a nanometer surface finish and remove the metal oxides and micro-cracks generated on the surface of laser-processed SLM-316L stainless steel. The grinding tool used to remove the metamorphic layer produced by the laser-processed surface is silicon carbide sandpaper with a grain size of 4000 (GSZ-C20, TRUER, Jiangsu, China). The grinding disk was rotating at 180 rpm, and the grinding time was 10 min. After mechanical grinding, the surface of laser-processed SLM-316L stainless steel became as smooth as a mirror (see Figure 8a–c). AFM measurement results show that the surface roughness of the three representative samples was 2.75, 2.95, and 3.41 nm, respectively, as shown in Figure 8d–f. This nanoscale surface finish is mainly attributed to mechanical polishing, not laser treatment, because laser polishing can only reduce the Sa value of SLM-316L stainless steel to 1.9 μm, while mechanical polishing can further reduce the Sa value to about 3 nm. Although laser processing cannot achieve precision polishing of stainless steel, it can modify the material and make it easier to remove by subsequent mechanical grinding. The micro-morphology of SLM-316L stainless steel (see Figure 9a) illustrates that the micro-cracks on the SLM-316L stainless steel surface disappeared after mechanical grinding. The XRD spectrum of SLM-316L stainless steel after grinding (see Figure 9b) shows three diffraction peaks at 2θ = 43.66°, 50.82°, and 74.72°, which are the same as the ones in Figure 1d. This indicates that the metal oxides were completely removed from the surface of laser-processed SLM-316L stainless steel.

## 4. Conclusions

In this paper, a nanosecond laser was used to rough polish SLM-316L stainless steel. Subsequently, the surface of the SLM-316L stainless steel was precisely polished by mechanical grinding. The main conclusions are as follows:

(1) The optimal laser processing parameters are 0.5 mm laser defocusing amount, 14 W laser power, 100 mm/s laser scanning speed, 8 μm laser scanning pitch, and 25 laser scanning times. Under optimal machining parameters, the surface roughness of SLM-316L stainless steel was reduced from 7.912 μm to 1.936 μm.
(2) Deteriorated layers appeared on the surface of the laser-treated stainless steel. EDS and XRD results indicate that the surface metamorphic layer consists of Fe_2_O_3_, Fe_3_O_4_, and Fe_1.67_Mo_1.33_O_4_. This is due to the oxidation reaction between the metal elements in SLM-316L stainless steel and the oxygen present in the air during the laser polishing process.
(3) The surface of SLM-316L stainless steel after laser processing became flat but had randomly distributed irregular micro-cracks. This phenomenon arises from the presence of inherent defects within the SLM-316L stainless steel. The defects will significantly increase the absorption of laser energy, resulting in a sharp rise in temperature and thermal stress at the defect. This induces the formation of micro-cracks at these defect sites.
(4) After undergoing laser treatment, the surface of the stainless steel underwent oxidation, resulting in the formation of metal oxides. Additionally, numerous micro-cracks, which were randomly distributed, appeared on the surface. These laser-induced surface alterations facilitated the subsequent mechanical grinding process. AFM measurements revealed that the surface roughness of stainless steel after mechanical grinding was about 3 nm. Moreover, the metal oxides and micro-cracks on the stainless steel surface were completely removed during this process.

## Figures and Tables

**Table 1:** Laser processing parameters and their values.

Factors	Level 1	Level 2	Level 3	Level 4
*H*/mm	0	0.5	1	1.5
*P*/W	8	10	12	14
*S*	15	20	25	30
*V*/(mm/s)	75	100	125	150
*D*/μm	8	10	12	14

**Table 2:** L_16_ (4^5^) orthogonal experiment table and the results of the surface roughness.

Expt. No.	*H*/mm	*P*/W	*S*	*V*/(mm∙s^−1^)	*D*/μm	Average Roughness/μm
1	0	8	15	75	8	4.003
2	0	10	20	100	10	2.749
3	0	12	25	125	12	2.630
4	0	14	30	150	14	2.637
5	0.5	8	20	125	14	6.596
6	0.5	10	15	150	12	3.673
7	0.5	12	30	75	10	3.502
8	0.5	14	25	100	8	1.936
9	1	8	25	150	10	6.178
10	1	10	15	125	8	7.259
11	1	12	30	100	14	7.123
12	1	14	20	75	12	2.657
13	1.5	8	30	100	12	5.745
14	1.5	10	25	75	14	7.337
15	1.5	12	20	150	8	5.671
16	1.5	14	15	125	10	5.898

**Table 3:** Range analysis results.

Parameter	*H*/mm	*P*/W	*S*	*V*/(mm∙s^−1^)	*D*/μm
*K*_1_	3.004	5.631	5.208	4.375	4.717
*K*_2_	3.297	5.255	4.418	4.388	4.582
*K*_3_	5.804	4.732	4.520	5.596	3.676
*K*_4_	6.163	3.282	4.752	4.540	5.923
*R*	3.159	2.349	0.79	1.221	2.247

**Table 4:** Variance analysis of surface roughness model.

Source	*SS*	*df*	*MS*	*F*	Reliability
*H*	12.793	3	4.264	0.230	Significant
*P*	9.099	3	3.033	0.162	Significant
*S*	1.462	3	0.487	0.026	
*V*	4.135	3	1.378	0.074	
*D*	10.206	3	3.402	0.184	Significant
Total	37.695	15			

**Table 5:** Material properties used for simulation.

Physical Properties of Materials	
Thermal conductivity	24.55 W/(m·K)
Density	7650 kg/m^3^
Constant pressure heat capacity	770.2 J/(kg·K)
Modulus of elasticity	193 GPa
Coefficient of thermal expansion	1.7 × 10^−5^ (1/K)

## Data Availability

Data is available upon reasonable request.
